# Job Flexibility, Job Security, and Mental Health Among US Working Adults

**DOI:** 10.1001/jamanetworkopen.2024.3439

**Published:** 2024-03-25

**Authors:** Monica L. Wang, Marie-Rachelle Narcisse, Katherine Togher, Pearl A. McElfish

**Affiliations:** 1 Department of Community Health Sciences, Boston University School of Public Health, Boston, Massachusetts; 2 Department of Health Policy and Management, Harvard T.H. Chan School of Public Health, Boston, Massachusetts; 3 Department of Psychiatry and Human Behavior, Brown University, Providence, Rhode Island; 4 College of Medicine, University of Arkansas for Medical Sciences Northwest, Springdale

## Abstract

**Importance:**

Understanding the association between job characteristics and mental health can inform policies and practices to promote employee well-being.

**Objective:**

To investigate associations between job characteristics and mental health, work absenteeism, and mental health care use among US adults.

**Design, Setting, and Participants:**

This cross-sectional study analyzed data from the 2021 National Health Interview Survey and included adults aged 18 years or older who reported employment during the past 12 months. Data were analyzed from May 2023 to January 2024.

**Exposures:**

Job flexibility was assessed as a summative variable to 3 questions: perceived ease of changing one’s work schedule to do things important to oneself or their family, regularity of work schedule changes, and advance notice of work hours. Job security was measured as perceived likelihood of losing one’s job.

**Main Outcomes and Measures:**

Mental health outcomes included self-reported serious psychological distress and frequency of anxiety. Work absenteeism was assessed using the number of missed workdays due to illness. Mental health care use was examined for both current and past year use. Multivariable logistic and binomial regression analyses were used to examine associations of interest.

**Results:**

The analytic sample consisted of 18 144 adults (52.3% [95% CI, 51.5%-53.2%] male; mean age, 42.2 [95% CI, 41.9-42.6] years). Greater job flexibility was associated with decreased odds of serious psychological distress (odds ratio [OR], 0.74 [95% CI, 0.63-0.86]; *P* < .001) and lower odds of weekly anxiety (OR, 0.89 [95% CI, 0.81-0.97]; *P* = .008) or daily anxiety (OR, 0.87 [95% CI, 0.79-0.96]; *P* = .005). Greater job security was associated with decreased odds of serious psychological distress (OR, 0.75 [95% CI, 0.65-0.87]; *P* < .001) and lower odds of anxiety weekly (OR, 0.79 [95% CI, 0.71-0.88]; *P* < .001) or daily (OR, 0.73 [95% CI, 0.66-0.81]; *P* < .001). Greater job flexibility (incidence rate ratio [IRR], 0.84 [95% CI, 0.74-0.96]; *P* = .008) and job security (IRR, 0.75 [95% CI, 0.65-0.87]; *P* < .001) were each associated with decreased number of days worked despite feeling ill over the past 3 months. Greater job security was associated with decreased absenteeism in the past year (IRR, 0.89 [95% CI, 0.82-0.98]; *P* < .014).

**Conclusions and Relevance:**

Organizational policies that enhance job flexibility and security may facilitate a healthier work environment, mitigate work-related stress, and ultimately promote better mental health.

## Key Points

**Question:**

How are job characteristics, such as job flexibility and job security, associated with employee mental health, work absenteeism, and mental health care use?

**Findings:**

In this cross-sectional study of 18 144 US adults who were employed, greater job flexibility was significantly associated with reduced odds of experiencing serious psychological distress and experiencing anxiety. Greater job security was significantly associated with reduced odds of experiencing serious psychological distress and experiencing anxiety.

**Meaning:**

These findings suggest that implementing workplace changes and policies that increase job flexibility and security may reduce work-related stress and facilitate improved employee mental health.

## Teaser

This cross-sectional study investigates associations between job characteristics and mental health, work absenteeism, and mental health care use among US adults.

## Introduction

Mental health concerns are prevalent among adults in the US. A 2022 report from the Substance Abuse and Mental Health Services Administration showed that 57.8 million adults (22.8%) aged 18 years and older experienced some form of mental illness in the last year; 19.4 million adults (7.6%) reported having concurrent substance use disorder and any mental illness; and 12.3 million adults (4.8%) seriously contemplated suicide.^1^ Data from 2023 indicate that one-third of US adults experienced anxiety and/or depression, with about 50% of young adults aged 18 to 24 years experiencing these symptoms.^2^

Job characteristics, such as job insecurity and limited flexibility, contribute to poor mental health.^3,4,5^ Studies demonstrate that experiencing job insecurity and unemployment is associated with onset of depressive symptoms,^4^ and work schedule instability increases likelihood of psychological distress and job absenteeism.^3^ Furthermore, the COVID-19 pandemic exacerbated job insecurity, particularly among employees with lower-wage positions.^6^ The US Census Bureau’s Household Pulse Survey revealed that there were 4.2 million fewer jobs in October 2021 than in February 2020, with low-wage jobs accounting for 59% of all jobs lost during that time period.^7^

Given that mental health is influenced by organizational characteristics, such as job flexibility and job security, examining the associations between these exposures and mental health may identify target areas of intervention to improve employee well-being. Job flexibility and job security can be modified through a number of organizational approaches, such as expanding flexible work schedules, providing remote or hybrid work options, enhancing tenure-based benefits, and investing in training and upskilling programs to develop and retain talent.^8,9^ This study used cross-sectional data from adults 18 years and older in the 2021 National Health Institute Survey (NHIS) and aimed to (1) examine associations between job characteristics (job flexibility and job security) and mental health (serious psychological distress and frequency of anxiety); (2) examine associations between job characteristics and work absenteeism; and (3) examine associations between job characteristics and mental health care use. We hypothesized that greater job flexibility and job security would be associated with (1) decreased odds of experiencing serious psychological distress and decreased frequency of anxiety; (2) decreased work absenteeism over the past 3 and 12 months; (3) decreased number of days worked (despite feeling ill) over the past 3 months; and (4) increased mental health care use (past year or current).

## Methods

This cross-sectional study followed the Strengthening the Reporting of Observational Studies in Epidemiology (STROBE) reporting guideline.^19^ Since the NHIS data are deidentified and public, the study was determined not to be human participant research and was exempted by the University of Arkansas Medical Services institutional review board. Informed consent was waived because data were deidentified and had been previously collected.

### Data Source

This study analyzed cross-sectional data from the NHIS 2021, which is an annual population survey.^10^ The NHIS includes data on health insurance, health care access, health services, health status, and sociodemographic characteristics. The NHIS uses a complex, multistage probability sampling technique with stratification and clustering designed to represent the civilian, noninstitutionalized US population living in the 50 states and the District of Columbia. The National Center for Health Statistics (NCHS), from the US Centers for Disease Control and Prevention, selects 1 adult aged 18 years and older from random households to gather information from face-to-face interviews. The NCHS also randomly selects 1 child younger than 17 years, and a parent or guardian answers questions about the child’s health. This study was based on the NHIS adult data file.

### Study Population

In 2021, 29 482 adults aged 18 years or older completed the interview. The analytical sample was restricted to adults who reported employment over the past 12 months (1751 individuals), which was captured by the question, “When was the last time you worked for pay at a job or business, even if only for a few days?” Response categories included: “within the past 12 months; 1 to 5 years ago; over 5 years ago; never worked.” Adults who did not report working in the past week (“Last week, did you work for pay at a job or business? Yes or No”) but worked within the past 12 months were included (16 461 individuals). The final analytic sample included data from 18 144 adults after exclusion of 68 pregnant participants. After sampling weights application, this sample corresponded to an estimated population of 168 084 992 US adults who were not incarcerated (Figure).

**Figure.**
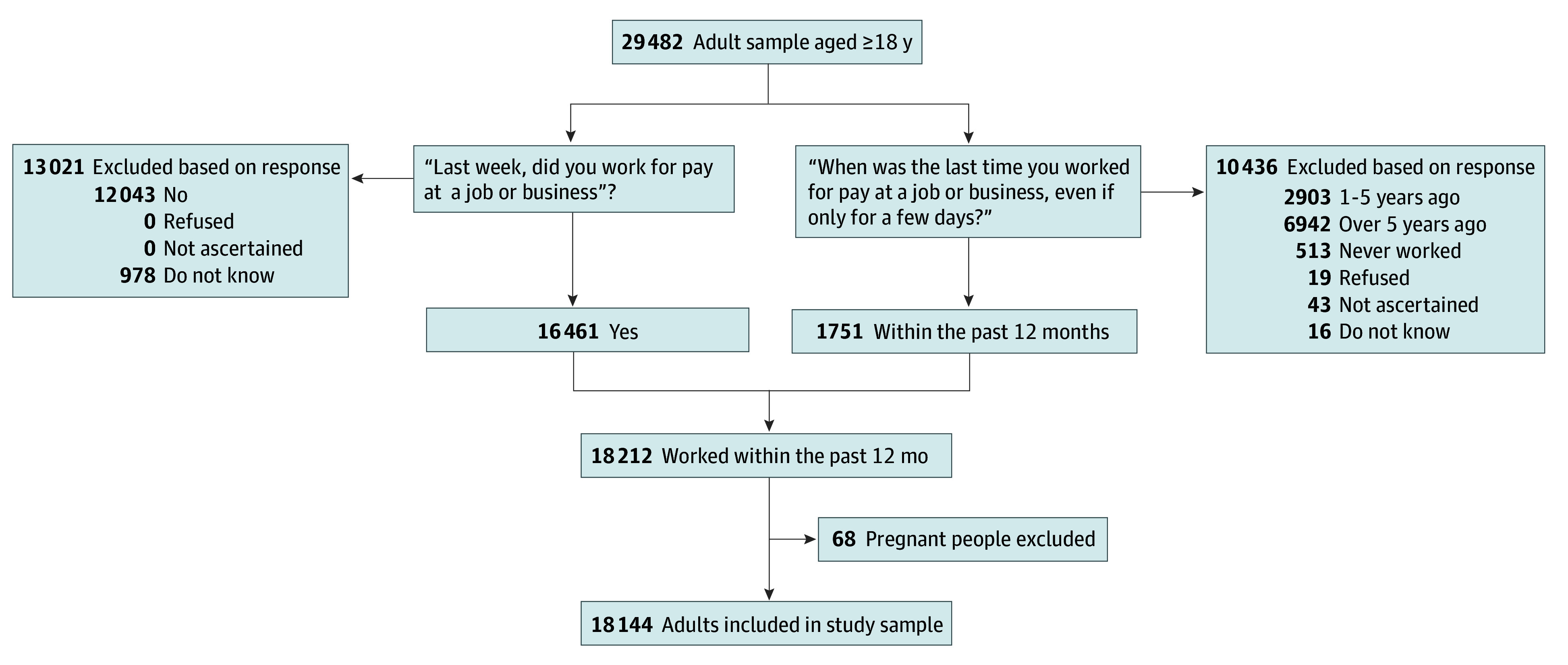
Participant Flow Diagram

### Measures

Three outcomes were analyzed: mental health (psychological distress and frequency of anxiety), work absenteeism, and mental health care use (current or past year). For mental health, psychological distress was measured by the Kessler-6, a 6-item questionnaire asking participants whether they experienced the following in the past 30 days: depression or sadness, nervousness, hopelessness, restlessness or fidgetiness, worthlessness, and feeling that everything was an effort.^11^ Each item is scored on a 5-point Likert scale (eg, 0, none of the time; 4, all of the time). Scores were summed to yield a Kessler-6 summative quasi-interval scale ranging from 0 to 24. Scores were dichotomized at the clinically validated cut point of 13 or more,^12^ classifying those with serious psychological distress (scores of 13 or more) or not (scores of 12 or less). Frequency of anxiety was captured by the question: “How often do you feel worried, nervous, or anxious? Daily, weekly, monthly, a few times a year, or never?”

The second outcome, work absenteeism, was captured by 3 continuous measures: (1) number of days missed while feeling ill over the past 3 months (0 to 90 days); (2) number of days worked while feeling ill over the past 3 months (0 to 90 days); and (3) number of days missed in the past 12 months (0 to 130 days or more). The third outcome, mental health care use, was captured by 2 dichotomous measures: (1) received mental health counseling over the past 12 months (yes or no) and (2) currently receiving mental health counseling (yes or no).

Two main exposures were analyzed: job flexibility, a summative variable based on the following questions: “How easy or difficult is/was it for you to change your work schedule to do things that are important to you or your family? Would you say very easy, somewhat easy, somewhat difficult, or very difficult?” (1, very or somewhat easy; 0, somewhat or very difficult); “Did your work schedule at your main job change on a regular basis?” (yes, 0; no, 1); “Approximately how far in advance do you usually know or does your employer usually tell you the hours you are scheduled to work on any given day?” (0, less than a week; 1, more than a week). The summative variable ranged from 0 to 3, with higher scores corresponding to greater job flexibility. The summative variable approach allowed us to conceptually capture a more comprehensive measure by combining questions that assess multiple aspects of work flexibility experiences and statistically mitigate potential challenges relating to multicollinearity of highly correlated individual variable items.

Job security was captured by the question: “Thinking about the next 12 months, how likely do you think it is that you will lose your job or be laid off?” Responses were reverse coded as 3 (not at all likely), 2 (somewhat likely), 1 (fairly likely), or 0 (very likely), with higher scores indicating higher job security. We examined both exposures continuously to capture a wide range of variation, allowing for a more nuanced exploration of their effects on outcomes across various levels of job flexibility and job security.

Covariates included biological sex (male or female), age, race or ethnicity, education, income (% federal poverty level), marital status, health insurance coverage, number of adults and children in the household, if they were born in the US, disability status, depression diagnosis (have you ever been told by a doctor or other health professional that you have depression; yes or no), and number of self-reported chronic health conditions. Race and ethnicity were self-identified. The race and ethnicity categories were predetermined by the NHIS and not determined by the investigators. Participants could identify as Hispanic, non-Hispanic American Indian or Alaskan Native, non-Hispanic American Indian or Alaskan Native and any other group, non-Hispanic Asian, non-Hispanic Black, non-Hispanic White, and other single or multiple races. Race and ethnicity were included in the study as covariates because both are associated with the exposures (job flexibility and job security) and are associated with the outcomes (mental health).

### Statistical Analysis

For descriptive analyses, we computed percentages for categorical variables and means for continuous variables. Bivariate analyses of each outcome by each of the exposure variables were performed. Adjusted Wald tests were used to determine statistically significant differences across exposure categories. Multicollinearity among independent variables (exposures and covariates) was assessed with the variance inflation indicators (VIF) and indicated no multicollinearity, as all VIF were less than 2.50 (mean [minimum-maximum] VIF, 1.34 [1.04-1.69]).^13^ Both unadjusted and adjusted regression analyses were conducted to account for potential influence of covariates.

To test our first hypothesis that greater job flexibility and job security are associated with decreased odds of experiencing serious psychological distress (adults without serious psychological distress as the reference group), we used multivariable logistic regression. For the categorical outcome of frequency of anxiety, we used multinomial logistic regression, with adults who reported never experiencing anxiety as the reference group.

The second hypothesis that greater job flexibility and security are associated with decreased work absenteeism over the past 3 and 12 months was tested using negative binomial (NB) regressions. For count outcome with overdispersed data (variance > mean) and excess zeros, NB regression is a more suitable technique than Poisson regression. The choice of NB vs zero-inflated NB (ZINB) was made based on epidemiological studies showing no significant difference between the NB and ZINB models, with results from NB being more straightforward to interpret than ZINB.^14,15^ Multivariable logistic regressions were conducted to test our third hypothesis that job flexibility and security were associated with increased odds of mental health care use.

The magnitude of the independent associations was gauged with odds ratios (ORs) for logit models and incidence rate ratios (IRRs) for count models along with 95% CIs. Statistical significance was a priori determined at α = .05. All descriptive and regression analyses accounted for the complex NHIS survey design (ie, final survey weights were used to compute unbiased estimates of descriptive parameters and regression parameters and design-based standard errors reflecting variance in the weights).^16^ Since NHIS imputed income data were used, missing data accounted for less than 5%; thus, complete case data analyses were conducted. We did not expect findings from regression analyses to change significantly due to low missingness.^17^ All analyses were performed with STATA SE version 17 (StataCorp).^18^ Data were analyzed from May 2023 to January 2024.

## Results

### Descriptive Analysis

#### Sociodemographic Characteristics of the Study Population

The study population included a diverse group of 18 144 participants, with males representing 52.3% (95% CI, 51.5%-53.2%) of the population and with a mean age of 42 (95% CI, 41.9-42.6) years. Participants identified as Hispanic (17.7% [16.4%-19.1%]), non-Hispanic Asian (6.2% [95% CI, 5.7%-6.8%]), non-Hispanic Black (11.4% [95% CI, 10.5%-12.4%]), non-Hispanic White (61.9% [95% CI, 60.2%-63.5%]), and other or multiracial (2.8% [95% CI, 2.3%-3.3%]).

#### Mental Health Profile

An estimated 3% of participants reported serious psychological distress, with 31.4% (95% CI, 30.6%-32.2%) experiencing anxiety symptoms a few times a year, 13.3% (95% CI, 12.7%-13.9%) monthly, 16.1% (95% CI, 15.5%-16.8%) weekly, and 12.2% (95% CI, 11.7%-12.8%) daily. Participants worked a mean of 2.0 (95% CI, 1.7-2.2) days while feeling ill over the past 3 months, missed 1.4 (95% CI, 1.3-1.5) days, and had a mean of 4.6 (95% CI, 4.3-4.9) days missed in the past 12 months. Additionally, 60.6% (95% CI, 58.0%-63.2%) were currently receiving mental health counseling whereas 11.4% (95% CI, 10.8%-12.0%) of those not currently in mental health counseling received such services in the past year (Table 1).

**Table 1.** Descriptive Statistics of the Study Population^a^

Characteristic	Weighted % (95% CI)
Mental health	
Experienced psychological distress^b^	
No	97.2 (96.9-97.4)
Yes	2.8 (2.6-3.1)
Frequency of anxiety	
Never	27.0 (26.1-27.9)
A few times a year	31.4 (30.6-32.2)
Monthly	13.3 (12.7-13.9)
Weekly	16.1 (15.5-16.8)
Daily	12.2 (11.7-12.8)
Work absenteeism, weighted mean (95% CI), d	
No. of days worked while feeling ill over the past 3 mos	2.0 (1.7-2.2)
No. of days missed while feeling ill over the past 3 mos	1.4 (1.3-1.5)
No. of days missed in the past 12 mos	4.6 (4.3-4.9)
Mental health care use	
Currently receiving counseling or therapy from mental health professionals	
No	39.4 (36.8-42.0)
Yes	60.6 (58.0-63.2)
Received counseling or therapy from mental health professionals, past 12 mos	
No	88.6 (88.0-89.2)
Yes	11.4 (10.8-12.0)
Exposures, weighted mean (95% CI)	
Job flexibility (0-3)	1.06 (1.05-1.07)
Job security (0-3)	2.78 (2.77-2.79)
Confounders	
Biological Sex	
Male	52.3 (51.5-53.2)
Female	47.7 (46.8-48.5)
Age, weighted mean (95% CI), y	42.2 (41.9-42.6)
Race or ethnicity	
Asian	6.2 (5.7-6.8)
Black	11.4 (10.5-12.4)
Hispanic	17.7 (16.4-19.1)
White	61.9 (60.2-63.5)
Other or multiracial^c^	2.8 (2.3-3.3)
Education	
Bachelor’s degree or less	59.5 (58.4-60.7)
More than a Bachelor’s degree	40.5 (39.3-41.6)
Marital status	
Single, divorced, or widowed	38.2 (37.3-39.1)
Married or partnered	61.8 (60.9-62.7)
Born in the US	
No	18.8 (17.7-20.0)
Yes	81.2 (80.0-82.3)
Household compositions, mean (SD)	
No. of adults in household	2.1 (2.1-2.1)
No. of children in household	0.7 (0.7-07)
Health insurance coverage	
No	9.7 (9.0-10.4)
Yes	90.3 (89.6-91.0)
Disability status	
Not disabled	96.2 (95.9-96.6)
Disabled	3.8 (3.4-4.1)
Income, % FPL	
<100	7.1 (6.6-7.8)
100-199	14.8 (14.1-15.5)
200-299	15.6 (15.0-16.2)
300-399	13.5 (12.8-14.1)
≥400	49.0 (47.9-50.2)
Ever diagnosed with depression	
No	84.7 (83.9-85.4)
Yes	15.3 (14.6-16.1)
No. of chronic conditions other than depression (0-10), mean (SD)	0.69 (0.68-0.71)

Abbreviation: FPL, federal poverty level.

^a^ Source: 2021 National Health Interview Survey—National Center for Health Statistics. Analytical study sample size (n = 18 114) and weighted sample size estimated at a representative population (N = 168 084 992) of adult civilians living in the United States.

^b^ Measured by Kessler-6. A score of 13 or more indicated experiencing psychological distress, and a score of less than 13 indicated not experiencing psychological distress.

^c^ Other includes non-Hispanic American Indian or Alaskan Native or any other race not listed.

### Bivariate Analysis

Adults with serious psychological distress (those with clinically validated scores of 13 or higher on the Kessler-6 scale) faced significantly lower job flexibility and job security ratings compared with their counterparts with lower distress levels (mean difference, 0.12 [95% CI, 0.04-0.20]; *P* = .003; mean difference, 0.23 [95% CI, 0.14-0.32]; *P* < .001, respectively). Those with daily anxiety had significantly lower job flexibility and job security ratings compared with their counterparts without anxiety (mean difference, 0.05 [95% CI, 0.01-0.10]; *P* = .022; mean difference, 0.11 [95% CI, 0.07-0.15]; *P* < .001, respectively).

Job flexibility ratings remained consistent regardless of how many days individuals missed work due to feeling ill in the past 3 months. However, adults with no absenteeism during this period reported significantly higher job security ratings (mean difference, 0.04 [95% CI, 0.01-0.07]; *P* = .02). Adults who abstained from working while feeling ill in the past 3 months experienced significantly higher job flexibility and job security ratings than their counterparts who worked at least 1 day while feeling ill during this period (mean difference, 0.10 [95% CI, 0.07-0.14]; *P* < .001; mean difference, 0.09 [95% CI, 0.06-0.12]; *P* < .001, respectively).

While job flexibility ratings were significantly higher among those who avoided missing any work days and those who missed at least 1 day in the past 12 months, job security ratings did not vary significantly between these 2 groups (mean difference, 0.09 [95% CI, 0.06 to 0.12]; *P* < .001; mean difference, −0.01 [95% CI, −0.03 to 0.01]; *P* = .497, respectively). There were no significant bivariate associations between any of the job characteristics and engagement in mental health counseling (current and throughout the past 12 months). See Table 2 for weighted means with corresponding 95% CIs and *P* values.

**Table 2.** Bivariate Associations Between Exposures and Outcomes^a^

Outcome	Exposures, weighted mean (95% CI)	
	Job flexibility	Job security
Mental Health		
Experienced serious psychological distress^b^		
Yes	1.07 (1.05-1.08)	2.79 (2.77-2.80)
No	0.94 (0.87-1.02)	2.56 (2.47-2.64)
Frequency of anxiety		
Never	1.09 (1.06-1.11)	2.79 (2.77-2.81)
A few times a year	1.05 (1.02-1.07)	2.81 (2.79-2.83)
Monthly	1.09 (1.05-1.14)	2.80 (2.77-2.83)
Weekly	2.80 (2.77-2.83)	2.76 (2.73-2.78)
Daily	1.03 (0.99-1.07)	2.68 (2.64-2.72)
Work absenteeism^c^		
No. of days missed while feeling ill over the past 3 mos		
0	1.07 (1.05-1.08)	2.79 (2.78-2.81)
≥1	1.04 (1.00-1.07)	2.76 (2.73-2.78)
No. of days worked while feeling ill over the past 3 mos		
0	1.08 (1.06-1.09)	2.80 (2.79-2.81)
≥1	0.98 (0.95-1.01)	2.71 (2.68-2.74)
No. of days missed in the past 12 mos		
0	1.10 (1.08-1.12)	2.78 (2.76-2.79)
≥1	1.01 (0.99-1.03)	2.78 (2.77-2.80)
Mental health care use		
Received counseling or therapy from mental health professionals in the past 12 mos		
No	1.06 (1.05-1.08)	2.78 (2.77-2.80)
Yes	1.07 (1.03-1.11)	2.75 (2.72-2.79)
Currently receiving counseling or therapy from mental health professionals		
No	1.09 (1.02-1.15)	2.78 (2.73-2.82)
Yes	1.06 (1.01-1.11)	2.74 (2.70-2.78)

^a^ Source: 2021 National Health Interview Survey—National Center for Health Statistics.

^b^ Measured by Kessler-6. A score of 13 or more indicated experiencing psychological distress, and a score of less than 13 indicated not experiencing psychological distress.

^c^ Work absenteeism variables measured in continuous days was dichotomized as 0 and ≥1 day.

### Regression Analysis

#### Associations Between Job Characteristics and Serious Psychological Distress

Greater job flexibility was associated with a 26% decrease in the odds of serious psychological distress (OR, 0.74 [95% CI, 0.63-0.86]; *P* < .001). Greater job security was associated with a 25% decrease of serious psychological distress (OR, 0.75 [95% CI, 0.65-0.87]; *P* < .001).

#### Associations Between Job Characteristics and Frequency of Anxiety

Job flexibility was associated with a 9% decrease in odds of experiencing anxiety a few times a year (OR, 0.91 [95% CI, 0.85-0.98]; *P* = .008) and an 11% decrease in the odds of experiencing weekly anxiety (OR, 0.89; [95% CI, 0.81-0.97]; *P* = .008). Job security was associated with a 21% decrease in the odds of weekly anxiety (OR, 0.79; [95% CI, 0.71-0.88]; *P* < .001).

Similarly, greater job flexibility was associated with a 13% decrease in the odds of daily anxiety (OR, 0.87 [95% CI, 0.79-0.96]; *P* = .005), while greater job security was associated with a 27% decrease in the odds of daily anxiety (OR, 0.73 [95% CI, 0.66-0.81]; *P* < .001).

#### Associations Between Job Characteristics and Absenteeism

As job flexibility increased, the estimated number of days absent (due to feeling ill) over the past 3 months increased, with change in work absenteeism increasing by 11% (IRR, 1.11 [95% CI, 1.01-1.22]). In contrast, as job security increased, short-term absenteeism decreased by 17% (IRR, 0.83 [95% CI, 0.73-0.94]; *P* = .004). As job flexibility and job security increased, the estimated number of days worked while feeling ill over the past 3 months decreased, corresponding to a decrease of 16% (IRR, 0.84 [95% CI, 0.74-0.96]; *P* = .008) and 25% (IRR, 0.75 [95% CI, 0.65-0.87]; *P* < .001). As job security increased, long-term absenteeism decreased by 11% (OR, 0.89 [95% CI, 0.82-0.98]; *P* = .02). Job flexibility was not associated with long-term work absenteeism (ie, 1 year).

Job flexibility was not significantly associated with past-year mental health counseling, whereas greater job security was associated with an 11% decrease in odds of receiving such counseling (OR, 0.89 [95% CI, 0.82-0.98]; *P* = .02). Job flexibility and job security were not significantly associated with receiving mental health counseling currently or in the past year (Table 3).

**Table 3.** Adjusted Associations Between Work Arrangements and Outcomes: Results From Multivariable Logistic Regression, Multinomial Logistic Regression, and Negative Binomial Regression^a^

Outcome	Odds ratio (95% CI)
Mental health	
Experienced psychological distress^b^	
No	1 [Reference]
Yes	
Job flexibility	0.74 (0.63-0.86)^c^
Job security	0.75 (0.65-0.87)^c^
Frequency of anxiety	
Never	1 [Reference]
A few times a year	
Job flexibility	0.91 (0.85-0.98)^d^
Job security	1.00 (0.91-1.09)
Monthly	
Job flexibility	0.96 (0.88-1.05)
Job security	1.00 (0.91-1.09)
Weekly	
Job flexibility	0.89 (0.81-0.97)^d^
Job security	0.79 (0.71-0.88)^c^
Daily	
Job flexibility	0.87 (0.79-0.96)^d^
Job security	0.73 (0.66-0.81)^d^
Work absenteeism, incidence rate ratio (95% CI)	
No. of days missed while feeling ill over the past 3 mos, d	
Job flexibility	1.11 (1.01-1.22)^e^
Job security	0.83 (0.73-0.94)^d^
No. of days worked while feeling ill over the past 3 mos, d	
Job flexibility	0.84 (0.74-0.96)^d^
Job security	0.75 (0.65-0.87)^c^
No. of days missed in the past 12 mos, d	
Job flexibility	0.95 (0.88-1.02)
Job security	0.89 (0.82-0.98)^e^
Mental health care use	
Receiving counseling or therapy from mental health professionals	
No	1 [Reference]
Yes, currently	
Job flexibility	1.06 (0.98-1.15)
Job security	0.89 (0.82-0.98)^e^
Yes, past 12 mos	
Job flexibility	0.97 (0.84-1.12)
Job security	0.90 (0.75-1.09)

^a^ Source: 2021 National Health Interview Survey—National Center for Health Statistics. Analytical study sample size (n = 18 114) and weighted sample size (N = 168 084 992) estimate of a representative population of adult civilians living in the United States. Job flexibility and job security are continuous variables ranging from 0 to 3. Odds ratios estimates for the outcome psychological distress and mental health care use are from multivariable logistic regression. Odds ratios estimates for the outcome frequency of anxiety are from multinomial logistic regression. Incidence rate ratios for the work absenteeism outcomes are from negative binomial regression.

^b^ Measured by Kessler-6. A score of 13 or more indicated experiencing psychological distress, and a score of less than 13 indicated not experiencing psychological distress.

^c^ Statistical significance: *P* < .001.

^d^ Statistical significance: *P* < .01.

^e^ Statistical significance: *P* < .05.

## Discussion

To our knowledge, this study is the first to evaluate the associations between mental health, work absenteeism, and mental health care use regarding job flexibility and job security among a nationally representative, random sample of US adults. Data from this study are from the 2021 NHIS, one of the largest national health surveys of adults in the country. Study findings indicated that greater job flexibility and higher job security were significantly associated with lower levels of psychological distress and less frequent feelings of anxiety among adults who were employed. Greater job flexibility was associated with higher work absenteeism (number of missed workdays due to illness) over the past 3 months, while greater job security was associated with decreased absenteeism over the past 3 and 12 months. Greater job flexibility and job security were both associated with a lower mean number of days worked despite feeling ill over the past 3 months. No association was found between job flexibility and security among participants who reported receiving mental health counseling (currently or over the past 12 months) compared with those who did.

Findings from this study are consistent with prior research on the association between mental health and job flexibility and job security. Increased job flexibility, such as reduced working hours,^20^ ability to work from home, or taking off time when needed,^8^ in addition to increased job stability have been shown to improve psychological outcomes and decrease anxiety among adults.^21,22,23^ Our study showed that greater job flexibility and job security were associated with 26% and 25% decreased odds, respectively, of experiencing serious psychological distress and a decrease in feelings of anxiety daily, weekly, and within the past year, indicating the substantive impact that flexible and secure jobs can have on mental health in the short-term and long-term. Increased job flexibility and job security can have positive outcomes on employees’ mental health for several reasons. Job flexibility, such as the ability to adjust work hours or work remotely, can help employees better balance work and personal life commitments and priorities.^8^ This can reduce stress and anxiety associated with managing competing demands, leading to improved mental well-being.^8,20,21,22,23^ Flexible job arrangements also allow employees to have more control over their schedules, making it easier to meet personal and family obligations. This balance can reduce burnout and psychological distress and promote better mental health.^3^

Our findings show mixed results regarding the association between job security and flexibility and work absenteeism. Higher job flexibility was associated with higher work absenteeism over the past 3 months, whereas higher job security was associated with less absenteeism over the past 3 and 12 months. The mixed findings likely arise from a complex interplay of multiple factors, such as different types of flexibility and security a job offers, individual priorities and needs, and workplace culture. Job flexibility can vary greatly across roles and industries; greater flexibility in remote work may lead to higher absenteeism if employees feel they cannot work from home when unwell, whereas hybrid or in-person roles (while still flexible in terms of scheduling) may be less conducive to employees taking sick leave. Job security may lead to lower work absenteeism due to higher work satisfaction, decreased job-related stress, and financial security.^4^ Employees with higher job satisfaction are less likely to experience mental health problems and absenteeism.^24^ The association between job flexibility, security, and absenteeism may also differ depending on individual needs, priorities, and workplace culture, as expectations on in-person presence and taking sick, medical, and personal time can vary greatly across organizations.

Job flexibility and job security were both associated with fewer mean number of days worked despite feeling ill over the past 3 months. This finding suggests that having flexible, secure jobs may encourage employees to prioritize their well-being and take sick leave and medical leave, rather than feeling pressured to work even when feeling ill. With flexible schedules, employees have greater flexibility in seeking health care,^25^ which can facilitate improved overall health in the long-term and result in fewer average sick days. A 2015 study conducted in England^26^ found small-sized and mid-sized enterprises that adopted job sharing or increased job security saw up to 6 fewer days of absence per year per employee.

Neither job flexibility nor job security were significantly associated with currently receiving mental health counseling, and only job security had any association with receiving mental health counseling during the past 12 months. The null findings may be due to limited or lack of mental health care coverage by employers through insurance policies, regardless of job security or flexibility. Additionally, while current job flexibility and job security may not influence immediate mental health care use, they may have a longer-term impact on employees’ ability to access mental health care services.

Findings from this study highlight several policies and practices that can be considered by organizations to enhance mental health among working adults. To promote job flexibility and allow employees greater control of work-life integration, flexible work schedules and remote or hybrid work options, work policies that enable adjustments in work hours to accommodate personal or family needs, and providing training and resources for managers and teams to effectively communicate and collaborate in flexible work arrangements can be implemented or enhanced.^8,27^ To increase job security, organizations can consider longer-term, flexible employee contracts^28^ and long-term strategies, such as skill development, uptraining, and career advancement opportunities.^4^ To improve health support, organizations can review and revise sick leave policies for short-term and longer-term absences, evaluate and expand mental health care coverage within employee benefit packages, and partner with clinicians or services that address access barriers, such as telehealth for mental health counseling.^3^

### Limitations

This study has limitations. For example, the cross-sectional survey design limits our ability to determine directionality. Mental health, absenteeism, and mental health care use was assessed via self-report and may be overestimated or underestimated. Study measures did not ask participants to specify if their anxiety, distress, absenteeism, or mental health care use was specific to job-related stressors. Dichotomizing psychological distress as an outcome limits our understanding of associations for other groups, such as individuals with moderate psychological distress. Job exposure and job flexibility were examined continuously, which assumes that the difference from each level of the variable is constant, an assumption that may not hold true in reality.

## Conclusions

Greater job flexibility and job security were associated with decreased serious psychological distress and lower anxiety among US working adults. These findings suggest that organizational policies that improve job flexibility and security may promote employee mental health and encourage use of mental health services when needed and ultimately improve overall employee well-being.

## Notes

Supplement.
Data Sharing Statement
