# Error in Grant Number in Funding/Support Section

**DOI:** 10.1001/jamanetworkopen.2026.25538

**Published:** 2026-07-02

In the Original Investigation titled “Job Flexibility, Job Security, and Mental Health Among US Working Adults,”^1^ published March 25, 2024, the grant number from the Community Engagement Alliance Against COVID-19 Disparities was incorrect. The grant number was originally listed as 10T2HL156812-01 and should have been listed as OT2HL158287. This article has been corrected.^1^
