# Supplementary material for: Job Flexibility, Job Security, and Mental Health Among US Working Adults
Source: JAMA Netw Open. 2024 Mar 25;7(3):e243439. doi: 10.1001/jamanetworkopen.2024.3439 (PMC10964112; doi:10.1001/jamanetworkopen.2024.3439)
Supplement: Supplement. — Data Sharing Statement [file jamanetwopen-e243439-s001.pdf]

Data Sharing Statement

Data
Data available: No

Additional Information
Explanation for why data not available: The 2021 National Health Interview Survey (NHIS) is openly made available by the Centers for Diseases Control and Prevention (CDC) at https://www.cdc.gov/nchs/nhis/2021nhis.htm. No datasets were generated for this study. Any analysis, interpretation, and/or conclusion based on the NHIS 2021 data is solely that of the authors. Opinions, conclusions, and recommendations expressed herein do not necessarily represent those of the National Center for Health Statistics or CDC, which are responsible for the data.
